# Mussel-inspired multi-bioactive microsphere scaffolds for bone defect photothermal therapy

**DOI:** 10.1016/j.mtbio.2024.101363

**Published:** 2024-11-23

**Authors:** Kaixuan Ma, Lei Yang, Wenzhao Li, Kai Chen, Luoran Shang, Yushu Bai, Yuanjin Zhao

**Affiliations:** a Department of Orthopedics, Shanghai Changhai Hospital, Naval Medical University, Shanghai, 200433, China; b Oujiang Laboratory (Zhejiang Lab for Regenerative Medicine, Vision and Brain Health), Wenzhou Institute, University of Chinese Academy of Sciences, Wenzhou, 325001, China; c Shanghai Xuhui Central Hospital, Zhongshan-Xuhui Hospital, and the Shanghai Key Laboratory of Medical Epigenetics, the International Co-laboratory of Medical Epigenetics and Metabolism (Ministry of Science and Technology), Institutes of Biomedical Sciences, Fudan University, Shanghai, 200032, China; d Department of Rheumatology and Immunology, Nanjing Drum Tower Hospital, School of Biological Science and Medical Engineering, Southeast University, Nanjing, 210096, China

**Keywords:** Bone regeneration, Hydrogel microsphere, Adhesion, Microfluidics, Tissue engineering

## Abstract

Hydrogel microspheres hold great promise as scaffolds for bone repair. Their hydrated matrix, biocompatibility, and functional properties make them an attractive choice in regenerative medicine. However, the irregularity of defect requires shape adaptability of the microspheres. Additionally, there is still room for improvement regarding the component of the microspheres to achieve sufficient bioactivity. Here, we prepare multi-bioactive microspheres composed of methacrylated silk fibroin (SFMA) *via* microfluidic electrospray. Magnesium ascorbyl phosphate (MAP) is encapsulated within the microspheres, whose sustained release facilitates angiogenesis and osteogenic differentiation. The microspheres are further coated with a polydopamine (PDA) layer, allowing them to assemble *insitu* into a scaffold that conforms to the non-uniform contours of bone defects. The photothermal conversion capability of PDA also provides mild photothermal stimulation to further promote bone regeneration. Based on the synergistic effects, our *in vivo* experiments demonstrated that the microsphere scaffold effectively promotes bone defect healing. Thus, this multi-bioactive scaffold offers a versatile strategy for bone repair with promising clinical potential.

## Graphical abstract

**Figure:**
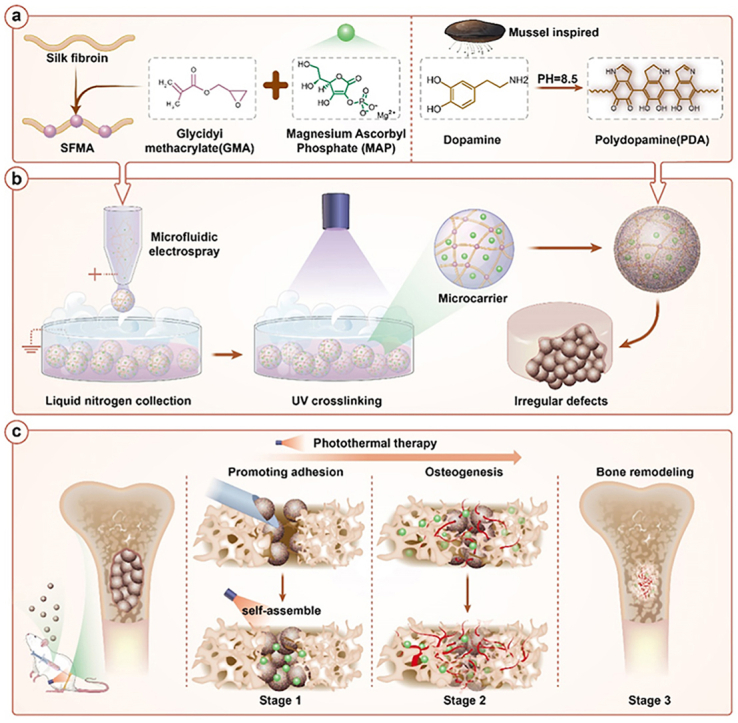

## 1 Introduction

Bone defect is a prevalent and complex orthopedic condition, which can arise from various factors, including trauma, infection, and bone tumor, leading to the loss of bone structural integrity [1,2]. Currently, the gold standard in clinical practice is bone grafting, which can be classified into autologous and allogeneic. However, the supply of autologous bone donors is limited, and allogeneic bone poses potential risks of rejection and pathogen transmission. Therefore, there is an urgent demand for developing bone graft substitutes [[3], [4], [5]]. The emergence of biomaterial scaffolds, especially hydrogel materials, has offered new prospects for bone repair, as their physicochemical properties can be tuned similar to natural extracellular matrix [[6], [7], [8], [9], [10]]. Thus, they not only avoid the problems of donor scarcity and rejection, but also facilitate endogenous bone regeneration, restoring the anatomical and functional integrity [11,12]. However, since the bone defect sites are often irregular, scaffolds without proper adhesion in the complex *in vivo* environment can lead to displacement and eventually repair failure [[13], [14], [15]]. In addition, synthetic material scaffolds often lack adequate bioactivity and require multi-step treatments with the addition of stimulatory agents to enhance osteogenic properties [16], which limit their biological effects and advanced functions [17,18]. Efforts are still needed to improve therapeutic outcomes by enhancing cellular responses such as adhesion, proliferation, and migration [19]. Therefore, novel bone scaffolds with good adhesion and bioactivity is still highly desired.

**Fig. 1:**
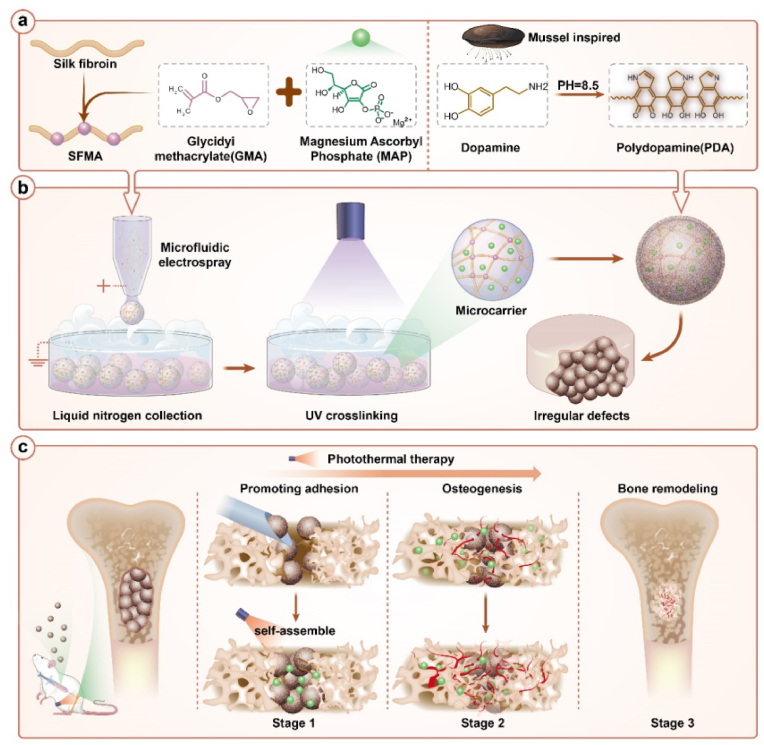
The schematic of mussel-inspired multi-bioactive microspheres for bone defect photothermal therapy. (**a**) The basic composition of microsphere scaffold, including SFMA, MAP, and PDA. (**b**) Fabrication of the hydrogel microspheres using electrospray, followed by UV cross-linking and self-assembly. (**c**) Application of the microspheres: they self-assemble into scaffolds *in situ* at bone defects. Upon photo-thermal agitation, the scaffold exhibits angiogenesis and osteogenesis activities, which promote bone defect repair.

Herein, we propose a mussel-inspired multi-bioactive microsphere scaffold for bone defect photothermal therapy, as shown in Fig. 1. Hydrogel microspheres can be fabricated with good monodispersity and injectability, making them suitable for filling in the defect site, with shape adaptability [20]. In particular, microspheres with appropriate adhesion features can assemble into stable scaffolds and better attach to the defect site. To achieve this, the mussel-inspired polydopamine (PDA) coating strategy is highly advantageous. On the other hand, natural-derived substances with biological activities have received increasing attention for composing biomaterial scaffolds [[21], [22], [23]]. Among these, magnesium ascorbyl phosphate (MAP), a natural ascorbic acid derivative, is a promising small molecule for promoting bone formation and osteogenic differentiation through various pathways [[24], [25], [26], [27], [28], [29]]. Therefore, by constructing microspheres combining the adhesion mechanism of PDA and the osteogenic characteristics of MAP, bioactive multifunctional bioactive microsphere scaffold can be developed for bone defect repair.

In this paper, we developed the desired hydrogel microsphere scaffolds through microfluidic electrospray technique. The hydrogel matrix of the microspheres was derived from natural silk fibroin (SF), with excellent biocompatibility, biodegradability, and mechanical strength [[30], [31], [32], [33], [34]]. Methacrylated silk fibroin (SFMA) was synthesized, and was mixed with MAP as the precursor. Droplets of the precursor were generated by electrospray and were rapidly frozen with liquid nitrogen, followed by photo-crosslinking under ultraviolet (UV) light, by which uniform-sized microspheres were fabricated. Subsequently, the surface of the microspheres was coated with PDA [[35], [36], [37]], which not only allowed the microspheres to assemble into scaffolds, but also endowed the microsphere scaffolds with photothermal responsiveness. The controlled release of MAP enhances reconstruction of blood vessels and promotes osteogenic differentiation. Additionally, mild photothermal stimulation was employed to further facilitate bone regeneration. With that, the microsphere scaffold exhibited excellent repair effects in a rat femoral defect model. Thus, we believe that this versatile scaffold offers a new strategy for bone repair and show promising application values.

## 2 Results and discussion

### 2.1 Fabrication of microspheres and their scaffold

**Fig. 2:**
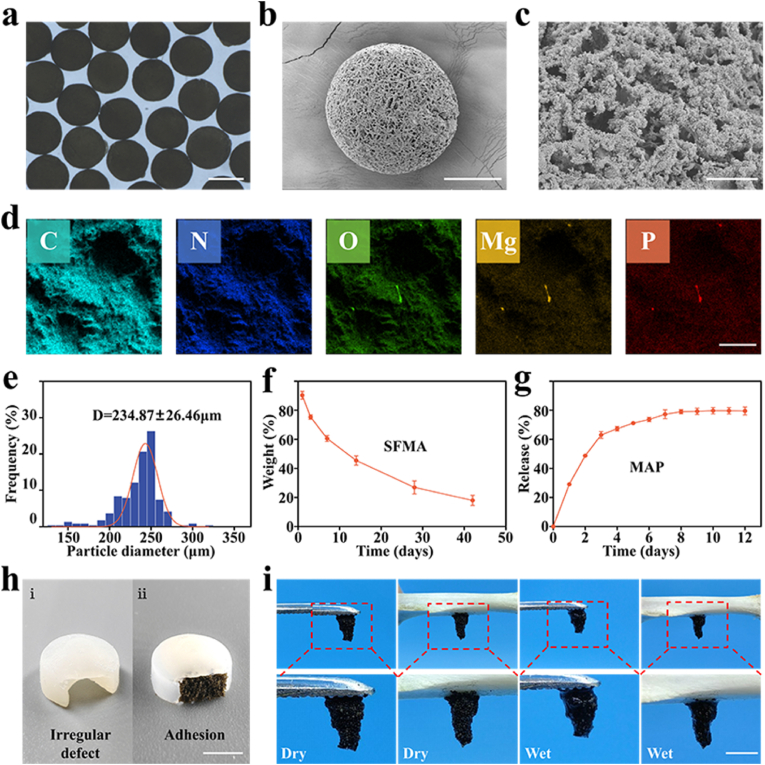
Characterization of the PDA-coated microspheres. (a)Morphology characterization of microspheres. (b, c) (b) Overall and (c) magnified SEM images of the porous structure of the microspheres. (d) Elemental analysis of the microspheres loaded with MAP. The scale bars in (a), (b), (c), and (d) are 200 μm, 100 μm, 10 μm, and 30 μm, respectively. (e) Size distribution of the microspheres. (f) Degradation curve, (g) release curve. (h) (hⅰ) Model of irregular defect, (hⅱ) microspheres self-assembled scaffold adhered well to irregular defect site, and (i) adhesion of the microsphere scaffolds to metal and femurs under dry and wet conditions. The scale bars in (h) and (i) are 0.5 cm.

In a typical experiment, microspheres were fabricated by microfluidic electrospray and further self-assembled into scaffolds. Initially, the pre-polymer solution of SFMA doped with MAP was subjected to electrospray *via* a microfluidic device (Fig. S1), and the resultant droplets were rapidly frozen in liquid nitrogen, followed by UV irradiation for photo-crosslinking. The as-prepared microspheres showed uniform spherical shape and porous structure (Fig. S2). In addition, the diameter of the microspheres varied negatively with the voltage and positively with the collection distance. These data demonstrated the tunability of this method (Fig. S3). To the endow microspheres with adhesion property, a mussel-inspired PDA coating procedure was carried out through mild chemical reaction. Upon completion of the reaction, the PDA-coated microspheres exhibited a black appearance **(**Fig. 2a, Fig. S4) as well as uniform spherical shape (Fig. 2a and b). The porous micromorphology of the microspheres was observed in the scanning electron microscope (SEM) images. (Fig. 2b and c). In addition, the PDA coating offered adhesion ability, allowing the spheres to assemble into an integral scaffold and adhere to various surfaces under both wet and dry conditions (Fig. 2h and i, Figure S5). Moreover, the elemental analysis indicated that Mg and P were present within the microsphere (Fig. 2d), further demonstrating the successful doping of MAP. In the *in vitro* degradation experiment, a PBS solution containing protease was used to simulate conditions at 37 °C. After 7 days, the microsphere scaffolds retained 60.59 ± 2.07 % of their mass. After 42 days, the retention rate was 17.98 ± 3.51 % (Fig. 2f). Furthermore, in the drug release experiment, approximately 80 % of MAP was released in the first week, and then (Fig. 2g) the release rate was more gently.

### 2.2 Photothermal response characteristics

**Fig. 3:**
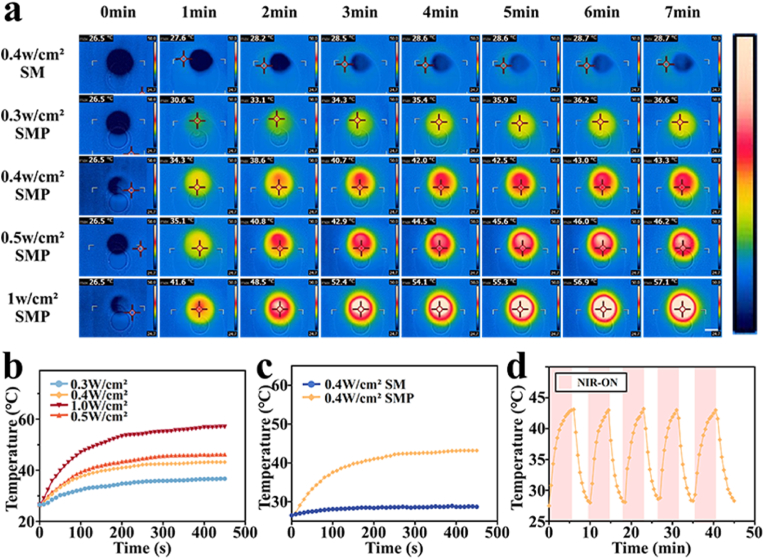
Photothermal responsiveness of microsphere scaffolds. (a–c) (a) Thermal images and (b, c) the associated thermal response curves. The scale bar in (a) is 1 cm. (d) The photothermal cycling curves of the microsphere scaffolds under NIR irradiation (0.40 W/cm^2^). The red area indicates when the NIR is activated during this period.

The photothermal behavior of SFMA/MAP@PDA microsphere scaffolds has been investigated using an 808 nm near-infrared (NIR) laser. Compared to SFMA/MAP microsphere scaffolds, SFMA/MAP@PDA microsphere scaffolds exhibit strong photothermal effects due to their efficient absorption of NIR, abbreviated as SM and SMP respectively. Under NIR irradiation, the temperature of the entire scaffold uniformly increased, and both the heating rate and equilibrium temperature was controlled by varying NIR power densities. Specifically, the temperature changes of SFMA/MAP@PDA scaffolds under 808 nm laser irradiation at 0.30, 0.40, 0.50, and 1.00 W/cm^2^ were monitored in real-time. The temperatures of each group gradually rose and stabilized at 36.6 °C, 43.3 °C, 46.2 °C, and 57.1 °C from a starting point of 27 °C (Fig. 3a and b). A comparison was also made between the temperature changes after 7 min of NIR irradiation at 0.40 W/cm^2^ on the scaffolds constructed with and without PDA coating; the temperature in the two groups increased from 26.5 °C to 43.3 °C and 28.7 °C, respectively (Fig. 3c). Since mild thermal stimulation (40–43 °C) has been proven to effectively promote bone regeneration, while excessively high temperatures may cause local tissue damage [38,39], we chose 0.40 W/cm^2^ for subsequent experiments. To demonstrate the repeatability of the photothermal effect (Fig. 3d), five photothermal cycles were completed by regulating the NIR on and off. In each cycle, the scaffolds were heated up to approximately 43 °C before recovering back to the initial temperature. The regular periodic curves indicated that the SFMA/MAP@PDA scaffolds possess good photothermal repeatability.

### 2.3 Promotion of angiogenesis and cell migration

**Fig. 4:**
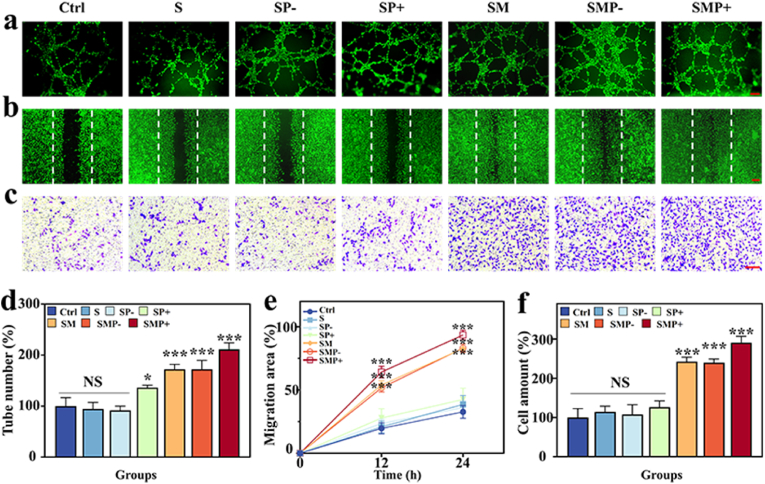
Evaluation of the angiogenesis and cell migration capability of the microsphere scaffolds *in vitro*. (a) Images of the tube formation assay. (b) Images of an *in vitro* scratch assay depict HUVECs, with dashed lines indicating the initial scratch edge. (c) Representative images of HUVEC transwell migration assay. Scale bars in (a), (b), and (c) are 20 μm, 200 μm, and 200 μm, respectively. (d) Quantitative analysis of tube formation. (e) Quantitative analysis of scratch assay of HUVECs *in vitro*. (f) Quantitative analysis of HUVEC transwell migration assay.

Effective vascularization of bone tissue is beneficial for osteogenesis [40,41]. To simulate the cell migration and angiogenesis in this process, we employed angiogenesis assays (Fig. 4a), scratch assays (Fig. 4b), and migration assays (Fig. 4c) with human umbilical vein endothelial cells (HUVECs). The groups were divided into Control, SFMA, SFMA@PDA (−), SFMA@PDA (+), SFMA/MAP, SFMA/MAP@PDA (−), and SFMA/MAP@PDA (+)**.** Abbreviated as Ctrl, S, SP-, SP+, SM, SMP-, and SMP+, respectively. In the groups containing PDA, the non-photothermal treatment groups were denoted with (−), and the photothermal treatment groups were denoted with (+). The *in vitro* tube formation assay demonstrated that treatment with the SM, SMP- and SMP + groups significantly enhanced the formation of a typical vascular network arrangement, offering precondition for early vascularization. Quantitative analysis revealed that at 6 h, the tube number in the SM, SMP- and SMP + groups significantly exceeded that in the Control, S, SP- and SP + groups (Fig. 4a–d). This indicated that the SFMA/MAP@PDA microsphere scaffold has a considerable ability to promote angiogenesis, accelerating the vascularization process. The scratch assay results indicate that the closure speed of the SM, SMP- and SMP + groups was significantly faster compared to the other groups (Fig. 4b–e). Additionally, the activity of MAP in enhancing cell migration rate was also confirmed in the transwell migration assay of HUVECs (Fig. 4c–f). Due to the effective release of MAP, the SM, SMP- and SMP + groups exhibited notably stronger migration activity than the other groups. Notably, the SMP + group exhibited enhanced tube formation, scratch repair, and cell migration abilities compared to the SMP- and SM groups. The SMP + group demonstrated strong functionality under the synergistic effects of MAP release and NIR thermal stimulation. This result is consistent with previous studies, which showed that mild thermal stimulation can enhance endothelial cell formation and migration capabilities [42,43].

### 2.4 Osteogenic activity

**Fig. 5:**
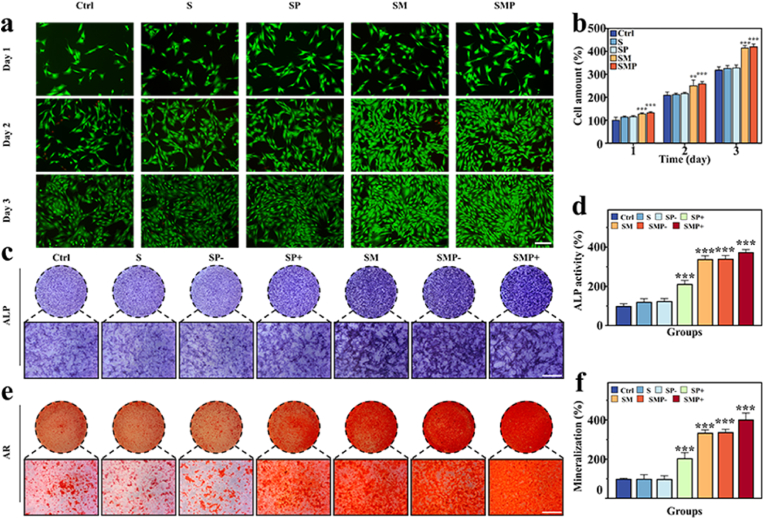
Biocompatibility and *in vitro* osteogenic activity of microsphere scaffolds. (a) Fluorescent images of rat BMSC co-cultured with microsphere scaffolds from different groups for 1, 2, and 3 days. Scale bar is 100 μm. (b) Cell viability of rBMSCs cultured for 1, 2, and 3 days using CCK-8 assay kit. (c) Representative images of ALP staining of rBMSCs cultured in medium containing microsphere scaffold for 14 days. Scale bar is 500 μm. (d) Relative ALP activity of different groups. (e) Representative images of AR staining of rBMSCs cultured in medium containing microsphere scaffold for 21 days. Scale bar, 500 μm. (f) Quantitative analysis of mineralization in different groups.

We next studied the proliferative and differentiation-enhancing activity of the microsphere scaffolds towards bone marrow stromal cells (BMSCs) *in vitro*. The groups included Ctrl, S, SP, SM, and SMP. In the proliferation assay, Calcein-AM staining results indicated that BMSCs maintained good cell viability and exhibited elongated spindle or fibroblast-like cell morphology after 3 days of culture (Fig. 5a). The Ctrl, S, and SP groups showed no significant differences throughout, demonstrating the materials’ excellent biocompatibility. Furthermore, compared to the aforementioned groups, the SM and SMP groups displayed higher cell viability within three days, suggesting that the doping of MAP significantly promoted the proliferation of BMSCs (Fig. 5a and b). Furthermore, we investigated the adhesion of cells to the microspheres before and after coating with PDA (Fig. S6). After co-culturing HUVECs with the microspheres, the HUVECs in the SMP group exhibited better spreading morphology and proliferation ability compared to the SM group. These results indicate that PDA-coated microspheres promote cells' adhesion behavior and cell growth [44].

In our subsequent investigation, we conducted *in vitro* differentiation assays. Specifically, we assessed the osteogenic differentiation of BMSCs using Alkaline Phosphatase (ALP) and Alizarin Red (AR) staining. After a two-week treatment, we observed that both the SM, SMP- and SMP + groups exhibited a more obvious ALP staining compared to the control group, S group, SP- group and SP + group, indicating higher levels of osteogenic activity (Fig. 5c and d). When BMSCs were treated for three weeks, the SMP + group formed more calcified mineral nodules than the other groups, and quantitative analysis further confirmed an increase in calcium accumulation on the scaffolds (Fig. 5e and f). These results suggested that the SMP + microsphere scaffolds are safe and efficient for bone repair, exhibiting biocompatibility, and promoting BMSCs proliferation and osteogenic differentiation. It also confirms that mild thermal stimulation provided by NIR irradiation promotes osteogenesis to some extent. Finally, bone formation was assessed at the genetic level using RT-QPCR. Similar to previous results, the SM, SMP-, and SMP + groups showed significantly increased expression levels of Runx2, ALP, and OCN compared to other groups, indicating that MAP plays a crucial role in upregulating the expression of osteogenic markers. Interestingly, compared to the SP- and SMP- groups, the SP+ and SMP + groups demonstrated enhanced induction of osteogenic marker expression (Fig. S7) [45].

### 2.5 *In vivo* bone defect repair

**Fig. 6:**
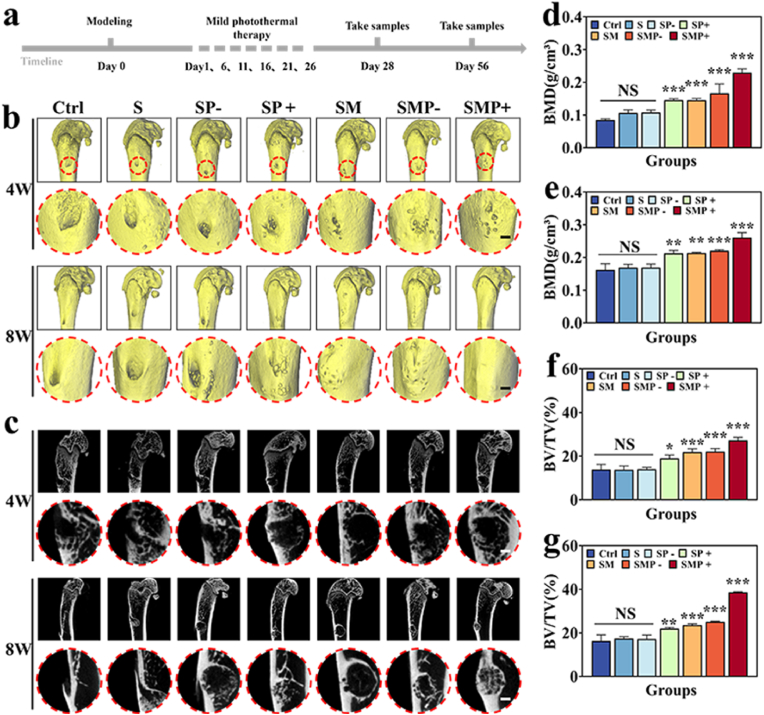
Analysis of osteogenesis *in vivo*. (a) Treatment procedure for the rat bone defect model. (b) 3D reconstruction images of the femur by Micro-CT. Scale bar is 1 mm. (c) Bone defects shown in the Micro-CT images. Bone defects caused by drilling are indicated by red circles. Scale bar is 1 mm. (d–g) Summary of the BMD at the (d) 4th and (e) 8th week, as well as the BV/TV at the (f) 4th and (g) 8th week. 4W and 8W represents the 4th and 8th week, respectively.

In the *in vivo* rat femoral defect model, the bone repair capabilities were assessed using microsphere scaffolds (Fig. 6a, Fig. S8). Initially, rats were divided into the following groups: Ctrl, S, SP-, SP+, SM, SMP-, and SMP+ (Fig. 6a), Subsequently, a 3 mm diameter femoral defect was created in each rat. Microspheres were then injected *in situ* and self-assembled into a scaffold in all groups except the control. Notably, the implanted microsphere scaffolds fitted well with the bone defect sites during the surgical process (Fig. S8). Moreover, under optimal experimental conditions, no diseases or deaths occurred among the rats throughout the experiment. In the photothermal groups, the rat femoral defect sites were irradiated with NIR every five days, with a total of six times. During the photothermal treatment, the temperature of the SFMA/MAP@PDA (+) hydrogel increased from 36 °C to 42.5 °C within 3 min (Fig. S9).

Femurs were collected at the 4th and 8th weeks, and the advancement of bone defect repair was tracked using animal micro-computed tomography (micro-CT), yielding three-dimensional reconstruction models and two-dimensional cross-sectional images (Fig. 6b and c). Among all groups at weeks 4 and 8, the SFMA/MAP@PDA (+) group exhibited the highest bone density (Fig. 6d and e) and bone tissue volume/total tissue volume (BV/TV) (Fig. 6f and g). Additionally, compared to the SFMA@PDA (−) and SFMA/MAP@PDA (−) groups, the SFMA@PDA (+) and SFMA/MAP@PDA (+) groups had higher BV/TV and bone density, further confirming that the photothermal effect of the scaffolds. Moreover, both at weeks 4 and 8, all groups containing MAP showed superior repair effects compared to the blank control group and those without MAP, which proves that MAP loaded in the scaffolds was successfully released and exerted its expected bioactivity, effectively promotes bone regeneration. These results collectively demonstrated that such multi-bioactive microsphere scaffolds with photothermal features can significantly enhance new bone formation *in vivo*, fill bone defects, and exhibit excellent repair capabilities.

### 2.6 Histological assessment

**Fig. 7:**
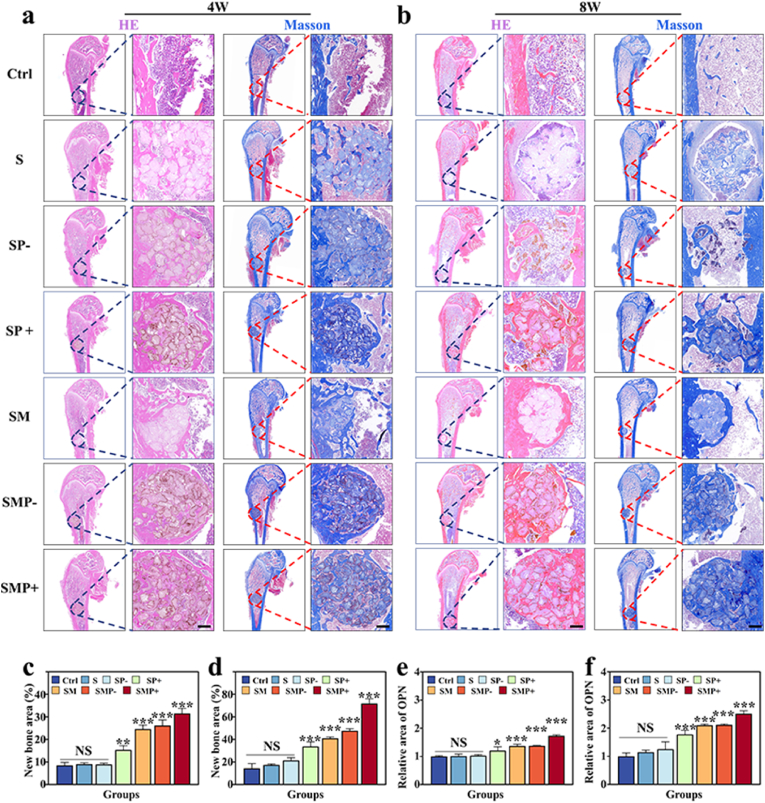
*In vivo* histological analysis. (a) Global and magnified views of Masson's trichrome and H&E staining of the femoral condyle at the 4th week post operation. Scale bar is 500 μm. (b) Global and magnified views of Masson's trichrome and H&E staining of the femoral condyle tissues at the 8th week post operation. Scale bar is 500 μm. (c, d) Quantitative analysis of defect size at the 4th and 8th weeks post operation. (e, f) Semi-quantitative IF analysis of OPN at the 4th and 8th weeks post operation.

In the histological assessment of the microsphere scaffolds *in vivo*, bone tissues were stained using Hematoxylin and Eosin (H&E) as well as Masson's trichrome. The results are depicted in Fig. 7a and b. For ease of observation, the defects were demarcated with dashed lines. The results revealed that in the control group, without scaffold support, only sparse bone tissue growth was present near the defect's edge. However, at weeks 4 and 8, H&E and Masson's staining demonstrated significant new bone formation around the bone defects in the SFMA/MAP, SFMA/MAP@PDA (−), and SFMA/MAP@PDA (+) groups (Fig. 7a–d), suggesting that MAP accelerated new bone formation. Notably, the photothermally treated groups, SFMA@PDA (+) and SFMA/MAP@PDA (+), exhibited more new bone tissue expanding from the defect's edge towards the center compared to their non-photothermally treated counterparts, SFMA@PDA (−) and SFMA/MAP@PDA (−), respectively. By week 8, the defect in the SFMA/MAP@PDA (+) group was almost filled, indicating the synergistic effect of both MAP and photothermal treatment.

**Fig. 8:**
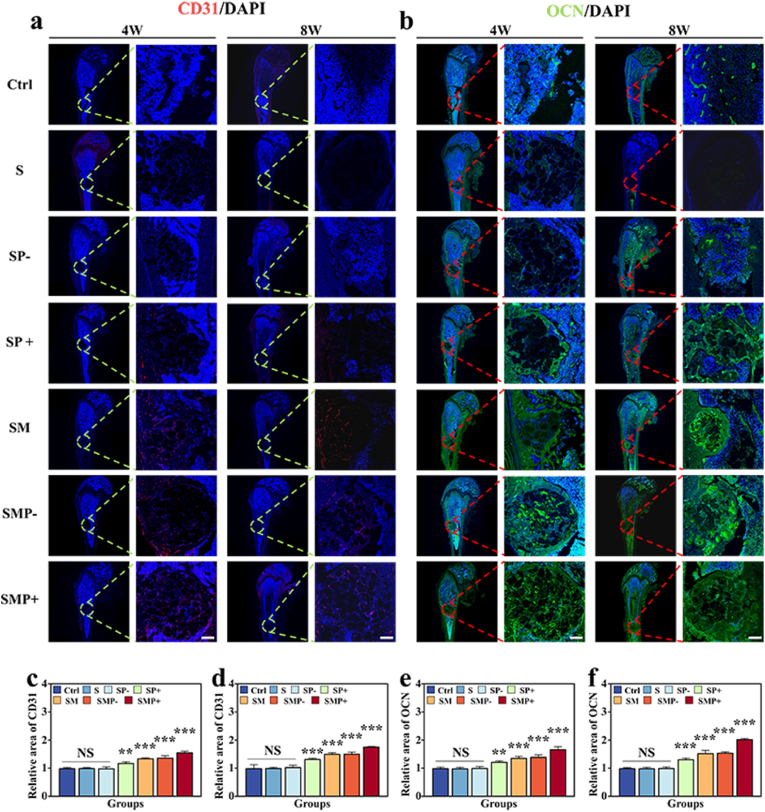
*In vivo* histological analysis. (a) IF staining of CD31 (red) in the defect area at 4 and 8 weeks postoperatively. Scale bar is 500 μm. (b) IF taining of OCN (green) in the defect area at 4 and 8 weeks postoperatively. Scale bar is 500 μm. (c–f) Semi-quantitative IF analysis of CD31 and OCN at 4 and 8 weeks postoperatively.

Moreover, immunofluorescence (IF) analysis for CD31, Osteopontin (OPN) and osteocalcin (OCN) was conducted at weeks 4 and 8 (Fig. 8a and b, Fig. S10). The SFMA/MAP@PDA (+) group exhibited considerably higher levels of CD31, OCN, and OPN compared to the other groups. The CD31 expression significantly increased in the MAP treated and photothermally treated groups (Fig. 8a–c, d). Additionally, in the SFMA/MAP@PDA (+) group, the expression levels of osteogenic cell markers, specifically OCN (osteocalcin) and OPN (osteopontin), were significantly increased. These findings suggest enhanced osteogenic activity (Fig. 7, Fig. 8b,e, f, Fig. S10). These results demonstrated that under the synergistic effect of MAP and photothermal action, the microsphere scaffolds can lead to the reformation of bone tissue in the defect area, accompanied by more new blood vessel formation, thereby productively enhancing the quality and speed of bone reconstruction.

## 3 Conclusions

In conclusion, we have developed a multi-bioactive microsphere scaffold endowed with excellent mussel-inspired adhesion and photothermal responsiveness, specifically engineered for bone defect repair. The material's synthesis is feasible, achieved through a simple process of electrospray and polydopamine coating. These resultant microspheres can then be suitably shaped to fit the defect site, self-assembling *in situ* into a scaffold that self-adheres. Such microsphere scaffolds exhibit good biocompatibility and appropriate physicochemical properties. The bioactive MAP loaded within the microsphere releases in a sustained manner, effectively promoting the formation of a vascular network at the defect site. Moreover, the excellent photothermal conversion capability of the microsphere scaffolds allows for controlled application of mild thermal stimulation to the bone tissue, further enhancing repair. *In vivo* experiments, including micro-CT and histological analyses, have demonstrated that the scaffolds show superior repair effects. This research, which achieves synergistic enhancement of repair efficiency through multiple mechanisms, can provide direction and considerations for the subsequent design of bone repair scaffolds [46]. For example, these microspheres can further carry seed cells and form a functionalized tissue engineering scaffold *in situ* [47]. Therefore, the present microsphere scaffold provides valuable guidance and feasible potential for the construction of multi-bioactive materials in bone defect repair.

## 4 Experimental section

*Materials****:*** MAP was sourced from Sigma (MO, USA). SFMA was synthesized in our laboratory according to established protocol [48]. PDA was purchased from Macklin (Shanghai, China). LAP was obtained from Aladdin Co. Ltd (Shanghai, China). All the antibodies were sourced from Servicebio Technology Co., Ltd (Wuhan, China).

*Microsphere preparation:* A 10 % solution of SFMA and MAP (1 mg/ml) was prepared in ultrapure water, followed by the addition of LAP (0.1 % v/v). The SFMA prepolymer and MAP blend underwent microfluidic electrospray, resulting in droplet formation under an applied electric field. These droplets were subsequently collected in liquid nitrogen, rapidly freeze-dried, and cured using UV light in a collection bath.

Following that, the microspheres were placed into a Tris-HCl (PH = 8.5) solution with dopamine (2 mg/ml) and were softly agitated for 8 h. The microspheres were then thoroughly washed with deionized (DI) water to obtain SFMA/MAP@PDA microspheres. Optical microscope images of the microspheres were obtained using a stereomicroscope. The surface morphology and structural features of the freeze-dried microspheres were analyzed using SEM (Sirion 200, FEI). Prior to use in cell and animal studies, the microspheres underwent sterilization by overnight exposure to 75 % ethanol followed by ultraviolet irradiation. Subsequently, they were rinsed with sterile PBS to remove the ethanol.

*Photothermal performance:* The photothermal properties of the microsphere scaffolds under 808 nm laser irradiation for 5 min were investigated. During irradiation, the laser was kept at a constant distance of 10 cm from the scaffold. An infrared thermal imaging camera was used to monitor real-time temperature increases induced by laser irradiation at various power densities (0.30, 0.40, 0.50, 1.00 W/cm^2^) and the corresponding thermal images. To test photothermal stability, the SFMA/MAP@PDA microsphere scaffolds were exposed to laser irradiation at 0.40 W/cm^2^. During each cycle, the sample was heated to approximately 43 °C and then allowed to return to the initial temperature. Laser on-off cycles were repeated 5 times to evaluate the repeatability and durability of the photothermal effect.

*In vitro drug release*: Microsphere scaffolds were immersed in a PBS solution. The absorbance of the solution was measured at the 261 nm absorption peak using a microplate reader. The release of MAP was quantified by comparison to a standard curve.

*In vitro biocompatibility:* BMSCs were divided into Control, SFMA, SFMA@PDA, SFMA/MAP, and SFMA/MAP@PDA groups. Following co-culturing for 1, 2, and 3 days in α-MEM supplemented with fetal bovine serum and penicillin-streptomycin, cell proliferation was evaluated using a CCK-8 assay. The absorbance was quantified using a microplate reader. Additionally, cells were stained with Calcein-AM to visualize their morphology, and the stained cells were examined using a fluorescence microscope.

*In vitro tube formation assay:* We used the Transwell co-culture model as described in Ref. [42] Briefly, 0.5 ml of each group of hydrogel microspheres were lyophilized, sterilized, and immersed in the upper chamber of the Transwell, with the cells placed in the bottom chamber, with or without NIR irradiation. HUVECs (5 × 10^4^ cells/well) were seeded onto the surface of a Matrigel-coated 24-well plate and divided into seven groups: Control, SFMA, SFMA@PDA (−), SFMA@PDA (+), SFMA/MAP, SFMA/MAP@PDA (−), and SFMA/MAP@PDA (+). After 6 h of incubation, HUVECs were stained with Calcein-AM and imaged using a fluorescence microscope. The total number of tubes formed was quantified and normalized to the control group.

*Scratch assays:* HUVECs (5 × 10^4^ cells/well, in a 24-well plate) were first incubated for 12 h. A sterile pipette tip was used to scratch each cell monolayer, and PBS was used to wash away unattached cells. The cells were divided into seven groups: Control, SFMA, SFMA@PDA (−), SFMA@PDA (+), SFMA/MAP, SFMA/MAP@PDA (−), and SFMA/MAP@PDA (+). The migration of HUVECs was recorded at specified time intervals.

*Transwell Migration Assay:* HUVECs (2 × 10^4^ cells/well) were placed in the upper chamber of the Transwell. Hydrogel microspheres from each group were placed in the lower chamber and incubated for 24 h. The upper chamber surface cells were wiped away with a cotton swab. Migrated cells were treated with 4 % paraformaldehyde and subsequently stained using 1 % crystal violet. Imaging was done with an inverted optical microscope.

*In vitro osteogenic differentiation:* BMSCs (2 × 10^4^ cells/well) were placed in the lower compartment of transwell chambers and were cultured in α-MEM. Upon reaching approximately 80 % confluence, the differentiation medium was substituted for the growth medium, and various types of microsphere scaffolds were placed in the top compartment of the transwell. After 14 and 21 days, we evaluated ALP and AR staining and activity following the manufacturer's instructions. Stained cells were imaged using an optical microscope to capture representative pictures. Quantitative analysis of ALP and AR activity was expressed as relative percentages compared to the control group.

*In vivo bone regeneration:* Male SD rats (approximately 190–240 g) were given by Vital River Laboratory Animal Technology Co., Ltd. (Zhejiang, China). All experimental animals received treatment strictly adhering to the guidelines for the care and use of laboratory animals. The experimental procedure received approval from the Animal Care and Use Committee of the Wenzhou Institute, University of Chinese Academy of Sciences, the approval number is WIUCAS23072001. During the initial four days, all rats were provided unrestricted access to water containing ceftriaxone sodium as a preventive measure against infection. Following the sterile saline rinse, we established a rat femoral defect model to evaluate the tissue regeneration capacity of the microsphere scaffolds *in vivo*. Specifically, a 3 mm depth and 3 mm diameter defect was surgically created at the distal femur using an electric drill. The rats were then randomly divided into Control, SFMA, SFMA@PDA (−), SFMA@PDA (+), SFMA/MAP, SFMA/MAP@PDA (−), and SFMA/MAP@PDA (+) groups. PBS solution containing different microspheres was injected into the femoral defect. The NIR group rats received repeated 808 nm laser irradiation (0.40 W/cm^2^, 5 min, 2 times) after implantation of the scaffold. The temperature was increased to approximately 43 °C during each session and maintained for 5 min. Then, the laser was turned off for 5 min. This cycle was repeated twice.

At the 4th and 8th week post-surgery, the femoral bone tissues were collected for macroscopic observation and Micro-CT analysis to assess bone regeneration. The scanning resolution was set at 18 μm. Images were acquired and cross-sectional images were reconstructed. From the reconstructed Micro-CT images, we quantified the BV/TV ratio and BMD. For histological examination, tissue samples were stained with HE and Masson's trichrome to assess femoral bone formation. Immunohistochemical staining was performed for OCN, OPN, and CD31 to study the angiogenesis and osteogenesis in the femoral defect.

*Statistical analysis:* Quantitative data were expressed as means ± standard deviations (SD). We employed one-way analysis of variance (ANOVA) to assess differences in experimental data across multiple groups and statistical significance set at ∗ p < 0.05, ∗∗p < 0.01, and ∗∗∗p < 0.001. The symbols mentioned above represent statistically significant differences between each experimental groups and the control group.

## CRediT authorship contribution statement

**Kaixuan Ma:** Writing – original draft, Visualization, Validation, Software, Resources, Methodology, Investigation, Data curation. **Lei Yang:** Supervision, Project administration, Methodology. **Wenzhao Li:** Writing – original draft, Investigation, Formal analysis, Data curation. **Kai Chen:** Validation, Project administration, Funding acquisition. **Luoran Shang:** Writing – review & editing, Writing – original draft, Project administration, Formal analysis. **Yushu Bai:** Writing – review & editing, Resources, Project administration, Funding acquisition, Conceptualization. **Yuanjin Zhao:** Writing – review & editing, Supervision, Resources, Project administration, Funding acquisition, Conceptualization.

## Declaration of competing interest

The authors declare that they have no known competing financial interests or personal relationships that could have appeared to influence the work reported in this paper.

## Data Availability

Data will be made available on request.
