# Supplementary material for: Mussel-inspired multi-bioactive microsphere scaffolds for bone defect photothermal therapy
Source: Mater Today Bio. 2024 Nov 23;29:101363. doi: 10.1016/j.mtbio.2024.101363 (PMC11629278; doi:10.1016/j.mtbio.2024.101363)
Supplement: Multimedia component 1 [file mmc1.docx]

**Supplementary**

**Mussel-inspired multi-bioactive microsphere scaffolds for bone defect photothermal therapy**

Kaixuan Ma ^1^, Lei Yang ^2^, Wenzhao Li ^2^, Kai Chen ^1,^*, Luoran Shang ^3,^*, Yushu Bai ^1,^*, Yuanjin Zhao ^1,4,^*

^1^ Department of Orthopedics, Shanghai Changhai Hospital, Naval Medical University, Shanghai 200433, China

^2^Oujiang Laboratory (Zhejiang Lab for Regenerative Medicine, Vision and Brain Health), Wenzhou Institute, University of Chinese Academy of Sciences, Wenzhou 325001, China

^3^ Shanghai Xuhui Central Hospital, Zhongshan-Xuhui Hospital, and the Shanghai Key Laboratory of Medical Epigenetics, the International Co-laboratory of Medical Epigenetics and Metabolism (Ministry of Science and Technology), Institutes of Biomedical Sciences, Fudan University, Shanghai 200032, China

^4^ Department of Rheumatology and Immunology, Nanjing Drum Tower Hospital, School of Biological Science and Medical Engineering, Southeast University, Nanjing 210096, China

Email: yjzhao@seu.edu.cn(Yuanjin Zhao); spinebaiys@163.com(Yushu Bai); luoranshang@fudan.edu.cn(Luoran Shang); ch_kai@163.com(Kai Cheng).


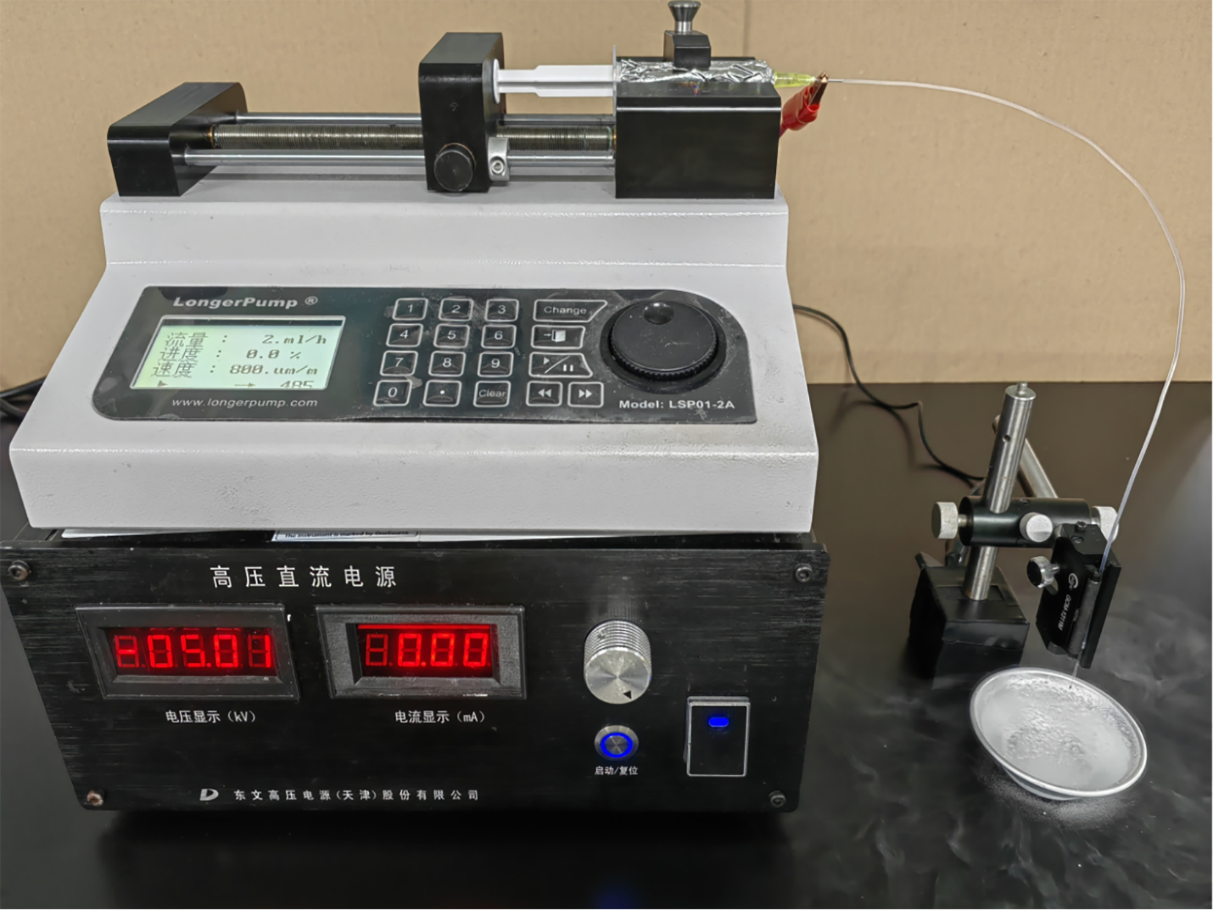


**Figure S1.** Photograph of the microfluidic electrospray setup used for microsphere generation.


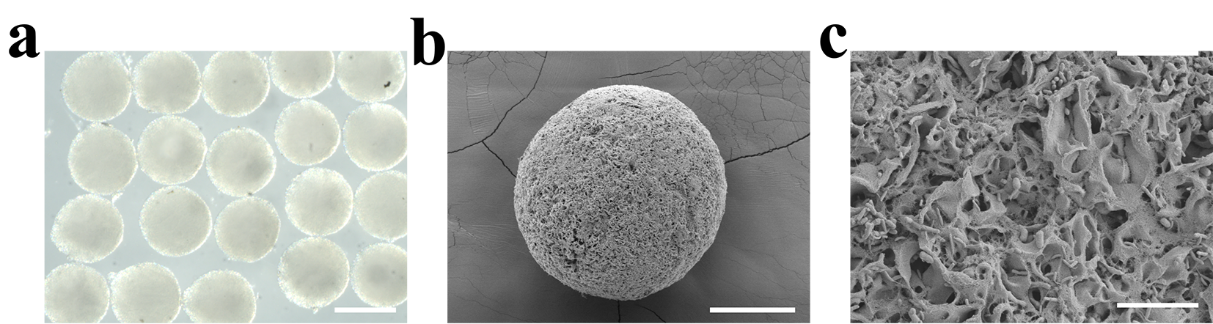


**Figure S2.** Characterization of the microspheres before dopamine coating. (a) Morphological characterization of microspheres. (b) SEM images showing the overall porous structure and (c) magnified view of the microspheres. The scale bars in (a), (b), and (c) are 200 μm, 100 μm, and 10 μm, respectively.


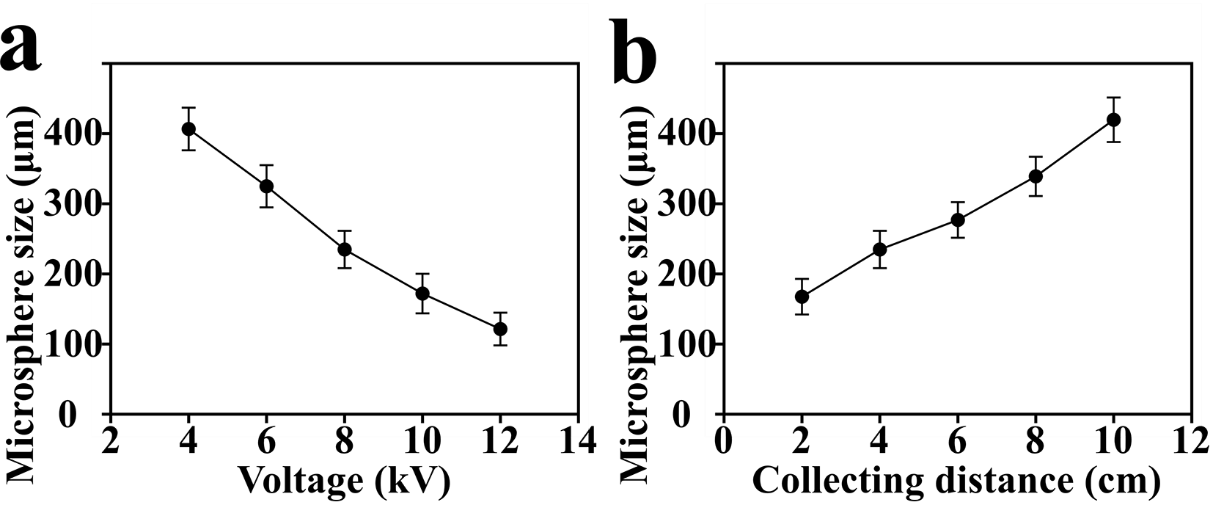


**Figure S3.** (a) The relationship between microsphere diameter and electrospray voltage in the microfluidic device. (b) The relationship between microsphere diameter and collection distance in the electrospray microfluidic device.


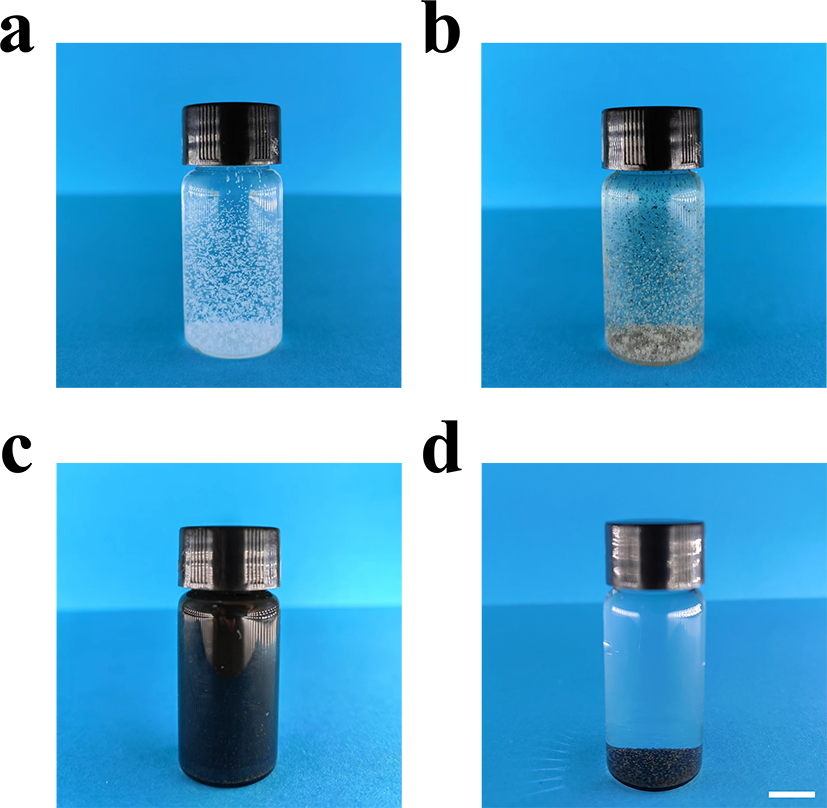
**Figure S4.** The process of dopamine coating. (a) Microsphere suspension before dopamine coating. (b) Microsphere suspension after 1 hour of dopamine coating. (c) Microsphere suspension after 8 hours of dopamine coating. (d) Microspheres after dopamine coating and subsequent washing with PBS for 3 times. Scale bar is 1 cm.


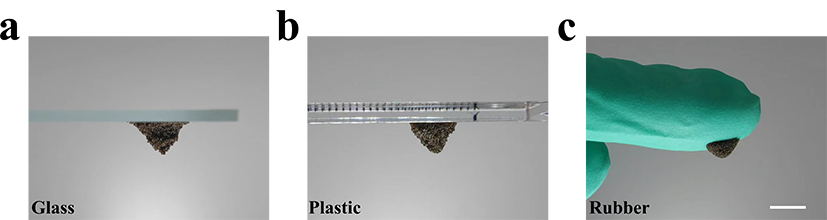
**Figure S5.**Microspheres self-assembled scaffold adhered to various surfaces. (a) Microspheres adhered to glass surface. (b) Microspheres adhered to plastic surface. (c) Microspheres adhered to rubber surface. Scale bar is 0.5 cm.


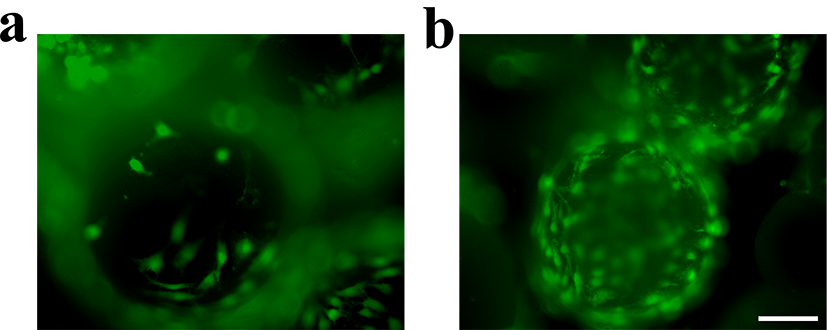
**Figure S6.** HUVECs co-cultured on hydrogel microspheres, (a) SM group (b) SMP group. The scale bar is 100 μm.


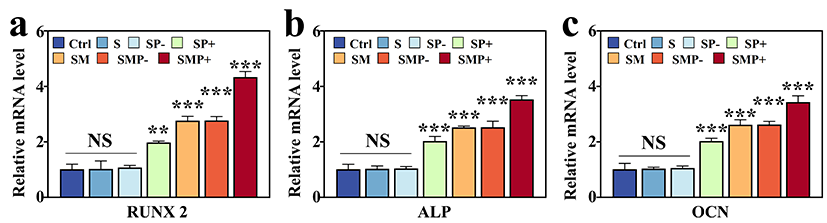
**Figure S7.** Relative mRNA expression of osteogenesis-related genes in BMSCs, including (a) Runx2, (b) ALP, and (c) OCN.


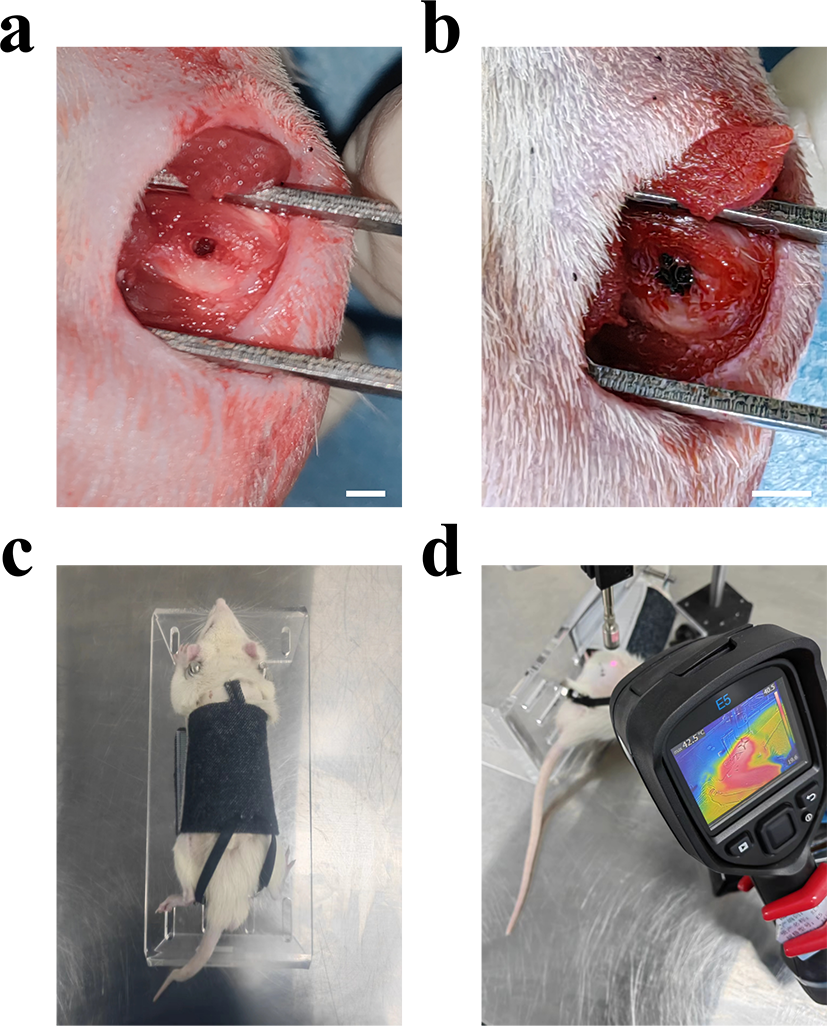
**Figure S8.**Animal experiment process. (a) Local magnification of rat femoral defect modeling. (b) Microspheres injected formed a scaffold and adhered well to the bone defect site. (c) Rat fixation device. (d) Process of photothermal therapy after rat femoral defect modeling. The scale bars in (a), (b) are 0.5 cm.


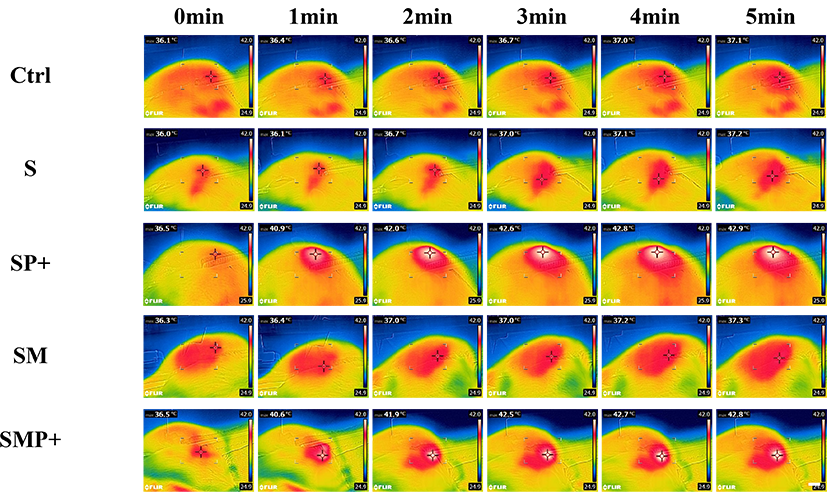
**Figure S9.** Thermal imaging of rat femurs from different groups under near-infrared irradiation. The scale bar is 0.5 cm


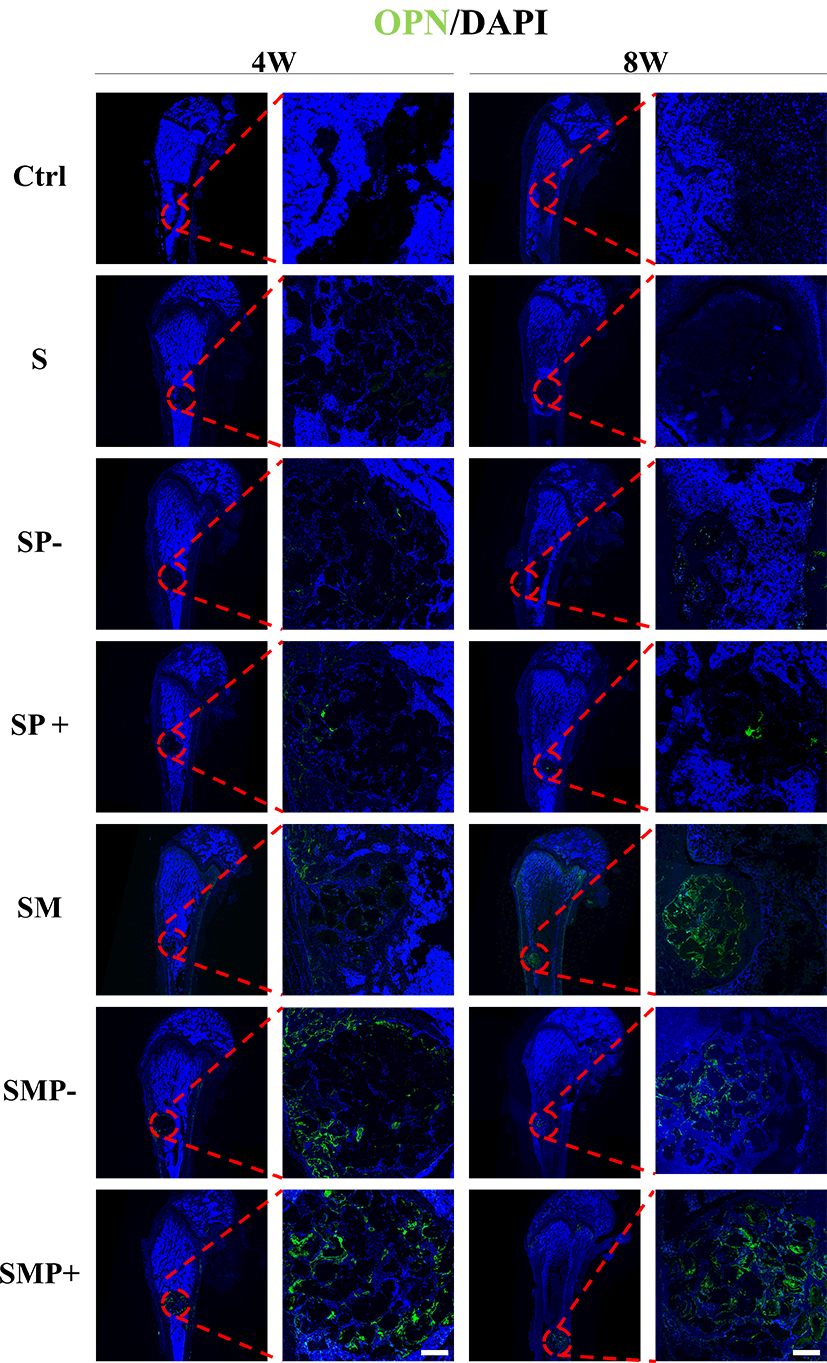
 **Figure S10.** IF staining of OPN (green) in the defect area the 4^th^ and 8^th^ weeks post operation. Scale bar is 500 µm.
